# Somatotropic axis drives bone turnover heterogeneity independently of gonadotropin synergy in girls with central precocious puberty

**DOI:** 10.3389/fendo.2026.1858179

**Published:** 2026-07-22

**Authors:** Fuhui Liu, Haiqin Jiang, Zhenzhen Li, Lei Li

**Affiliations:** 1 School of Clinical Medicine, Shandong Second Medicine University, Weifang, China; 2 Department of Geriatrics I, Weifang People's Hospital, Weifang, China; 3 Department of Pediatrics, Weifang People’s Hospital, Weifang, China

**Keywords:** bone turnover markers, central precocious puberty, insulin-like growth factor I, luteinizing hormone, somatotropic axis

## Abstract

**Background:**

The classic paradigm of somatotropic-gonadal axis synergy in pubertal bone acquisition lacks validation in central precocious puberty (CPP), and the driver of the significant bone turnover marker (BTM) heterogeneity in this population is unknown.

**Objective:**

To investigate the independent and potential synergistic associations of insulin-like growth factor-1 (IGF-1) and peak luteinizing hormone (LH) with BTMs in girls with CPP.

**Methods:**

This retrospective cohort included 190 treatment-naïve girls with CPP. Participants were stratified into Low, Medium, and High bone turnover groups based on tertiles of a bone age-standardized composite Z-score of bone gla protein (BGP) and β-C-terminal telopeptide (β-CTX). We used multiple linear regression adjusted for chronological age, supplemented by E-value analysis.

**Results:**

Marked BTM heterogeneity was confirmed (*p* for trend < 0.001). IGF-1 and peak LH levels exhibited significant graded increases across groups (*p* for trend = 0.003 and 0.030). After age-adjustment, IGF-1 independently correlated with both BGP (*β* = 0.069, *p* = 0.005) and β-CTX (*β* = 0.001, *p* = 0.030), while peak LH associated only with BGP (*β* = 0.411, *p* = 0.026). Critically, the IGF-1 × LH peak interaction was non-significant (*p* = 0.655 and 0.791 for BGP and β-CTX, respectively). In contrast, glucolipid metabolic parameters showed no differences, arguing against their major role and highlighting neuroendocrine specificity.

**Conclusion:**

In this cross-sectional study of girls with CPP, heterogeneity in bone turnover was more strongly and consistently associated with the somatotropic axis (IGF-1) than with gonadotropin activity, and no significant synergy was observed. These findings suggest a potential shift toward a somatotropic-dominant mechanism in the context of pathological pubertal acceleration, highlight IGF-1 as a pivotal biomarker for skeletal metabolism assessment, and challenge the classical paradigm of somatotropic-gonadal axis collaboration.​ The proposed mechanism requires validation in longitudinal studies.

## Introduction

Puberty constitutes a critical window for skeletal accrual, accounting for approximately 40-50% of peak bone mass acquisition (1). This process is classically described as being orchestrated by a synergistic interplay between the somatotropic and gonadal axes (2, 3). However, the validity of this paradigm in pathological states of accelerated puberty, such as central precocious puberty (CPP), remains unexamined. CPP, driven by premature activation of the hypothalamic-pituitary-gonadal (HPG) axis, offers a unique model to study bone regulation under conditions of elevated gonadal steroids (4, 5). The core pathogenic mechanism involves estrogen acting on the growth plate to accelerate chondrocyte differentiation and premature epiphyseal fusion, ultimately impairing adult height (6–8). Emerging evidence has further revealed that girls with CPP frequently exhibit distinct metabolic abnormalities, including elevated triglycerides and reduced high-density lipoprotein cholesterol (9–11). Notably, a bidirectional regulatory crosstalk exists between the skeletal and metabolic systems: bone-derived factors modulate glucose and lipid metabolism (12–14), while lipid-derived molecules reciprocally influence bone remodeling (15). This “bone-metabolism axis” provides a theoretical framework for explaining the coexistence of metabolic disturbances and accelerated skeletal maturation in CPP (16–18).

Clinically, significant heterogeneity in bone turnover markers (BTMs) has been observed among girls with CPP, but the underlying drivers remain elusive (19, 20). Is this heterogeneity dominated by metabolic factors, excessive activation of the gonadal axis, or independent regulation by the somatotropic axis? Disentangling the relative contributions of metabolic and neuroendocrine factors is critical for elucidating the regulatory mechanisms of bone metabolism in CPP.

Therefore, this study aimed to elucidate the drivers of bone turnover heterogeneity in a cohort of treatment-naïve girls with CPP. We quantified the independent and synergistic contributions of the somatotropic (as assessed by insulin-like growth factor-1 [IGF-1]) and gonadal (as assessed by peak luteinizing hormone [LH]) axes, thereby directly testing the applicability of the classic “dual-axis synergy” paradigm in this pathology. Furthermore, to assess whether the observed heterogeneity could be alternatively explained by alterations in general metabolic status, we evaluated the association of glucolipid metabolic parameters with bone turnover phenotypes.

## Methods

### Study design and participants

This is a retrospective, cross-sectional study.​ The study was approved by the Institutional Review Board of Weifang People’s Hospital (Approval No. KYLL20250821-7), with a waiver for informed consent. All clinical and biochemical data were collected at the single time point of the participants’ initial diagnosis of CPP, with no subsequent follow-up.

CPP was diagnosed by a positive gonadotropin-releasing hormone (GnRH) stimulation test, defined as a peak LH level >5.0 IU/L combined with a peak LH/peak follicle-stimulating hormone (FSH) ratio >0.6 (21). Supportive criteria included the onset of secondary sexual characteristics before 7.5 years of age and a bone age (BA) advancement of ≥1 year beyond chronological age (CA) (21).

**Figure 1:**
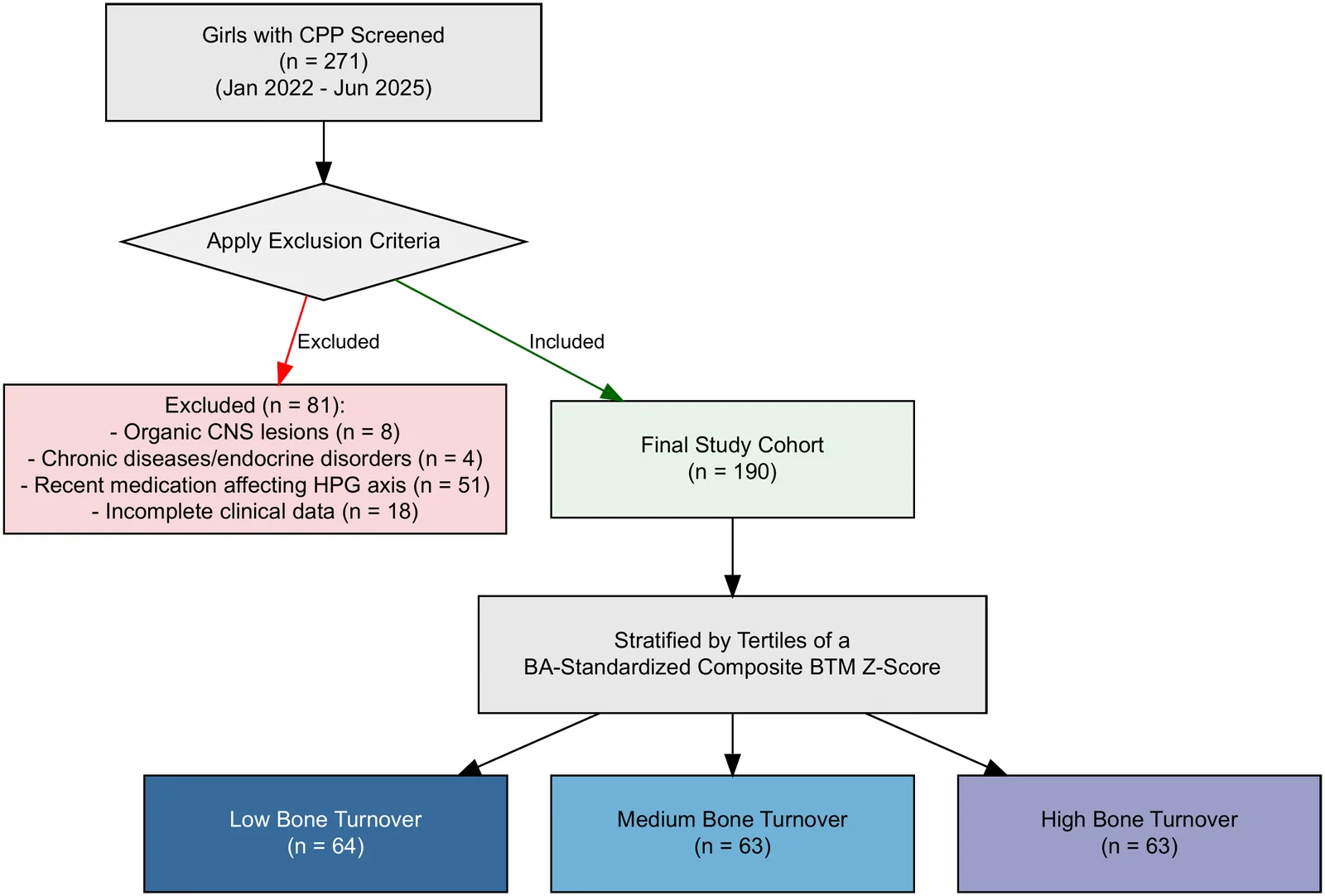
Flowchart of study participant selection. CNS,​ central nervous system; HPG axis, hypothalamic-pituitary-gonadal axis; BA,​ bone age; BTM, bone turnover marker.

We screened the medical records of 271 girls diagnosed with CPP at our department between January 2022 and June 2025. The process of applying the following exclusion criteria to this cohort is detailed in **Figure 1**:​ (1) organic central nervous system (CNS) lesions; (2) systemic diseases, diabetes, thyroid dysfunction, or tumors; (3) a history of using medications known to affect the HPG axis within the preceding 6 months; and (4) incomplete data for key analysis variables (specifically, IGF-1, peak LH, bone gla protein [BGP], or β-C-terminal telopeptide of type I collagen [β-CTX]) (22). After applying these criteria, 190 treatment-naïve girls were included in the final analysis.

Furthermore, to maintain a homogeneous cohort focused on the pathophysiology of activated HPG axis, girls with isolated premature thelarche who were evaluated during the same period were not included in this analysis.

### Clinical and biochemical assessments

All evaluations were performed at the initial diagnostic work-up. Following an overnight fast, venous blood samples were collected between 8:00 and 9:00 AM. The GnRH stimulation test was conducted using intravenous gonadorelin (2.5 μg/kg, maximum 100 μg). Blood samples for LH and FSH measurement were obtained at 0, 30, 60, and 90 minutes (23, 24). Serum levels of basal and peak LH/FSH, estradiol (E2), IGF-1, and insulin-like growth factor binding protein-3 (IGFBP-3) were measured using chemiluminescent immunoassays (Beckman Coulter DxI 800) (21, 25, 26). BTMs (BGP and β-CTX) were quantified by electrochemiluminescence immunoassay using a cobas e601 module​ (Roche Diagnostics, Basel, Switzerland) (27). Fasting blood glucose (FBG) and lipid profiles, including total cholesterol (TC), triglycerides (TG), high-density lipoprotein cholesterol (HDL-C), and low-density lipoprotein cholesterol (LDL-C), were analyzed using standard enzymatic methods. BA was assessed from a left hand-wrist radiograph by an experienced pediatric endocrinologist blinded to the biochemical results, using the Greulich-Pyle atlas method (28). Bone turnover markers (BGP and β-CTX) were measured as part of the routine initial diagnostic work-up for girls suspected of having CPP in our clinic.​ Pubertal staging (Tanner stage) was not systematically documented in the medical records for all participants and therefore could not be included in the analysis.

### Definition of bone turnover groups

To investigate heterogeneity in bone metabolism, participants were stratified based on BTMs. As skeletal maturation is the primary driver of bone turnover in CPP, BGP and β-CTX levels were standardized using BA-specific Z-scores, calculated as (individual value – cohort mean)/cohort standard deviation (29). A composite bone turnover score was derived by summing the Z-scores of BGP and β-CTX. Participants were then divided into tertiles based on this composite score, defining Low, Medium, and High bone turnover groups.

### Statistical analysis

All statistical analyses were performed using R software (version 4.5.2). Continuous variables are presented as mean ± standard deviation or median (interquartile range) based on the normality of distribution, which was assessed using the Shapiro-Wilk test. Categorical variables are summarized as frequencies (%). Differences across the three bone turnover groups were compared using one-way ANOVA, Kruskal-Wallis tests, or chi-square tests, as appropriate. The Jonckheere-Terpstra test was used to assess significant trends across the ordered groups.

We constructed two core multiple linear regression models to evaluate associations between endocrine factors and bone turnover markers (with BGP or β-CTX as the dependent variable). Model 1 examined the independent effects of IGF-1 (or IGFBP-3) and the LH peak, adjusted for chronological age. We specifically adjusted for chronological age rather than bone age to avoid circular reasoning, as bone age was intrinsically used to standardize the bone turnover markers for the creation of the exposure groups.​ Model 2 included an interaction term (IGF-1 × LH peak) to test for synergy. To avoid multicollinearity, IGF-1 and IGFBP-3 were analyzed in separate models.

Given the lack of data on body mass index (BMI) and body composition, which are potential confounders of bone metabolism, an E-value analysis was performed to assess the robustness of the significant associations to such unmeasured confounding (30). Although no *a priori* sample size calculation was performed for this exploratory study, the final sample of 190 participants allowed detection of statistically significant associations in the primary regression models.

Exploratory analyses included assessing correlations between BTMs and glucolipid metabolic parameters using Spearman’s rank correlation. A two-sided *p*-value < 0.05 was considered statistically significant.

## Results

### Study population and confirmation of bone turnover heterogeneity

**Figure 2:**
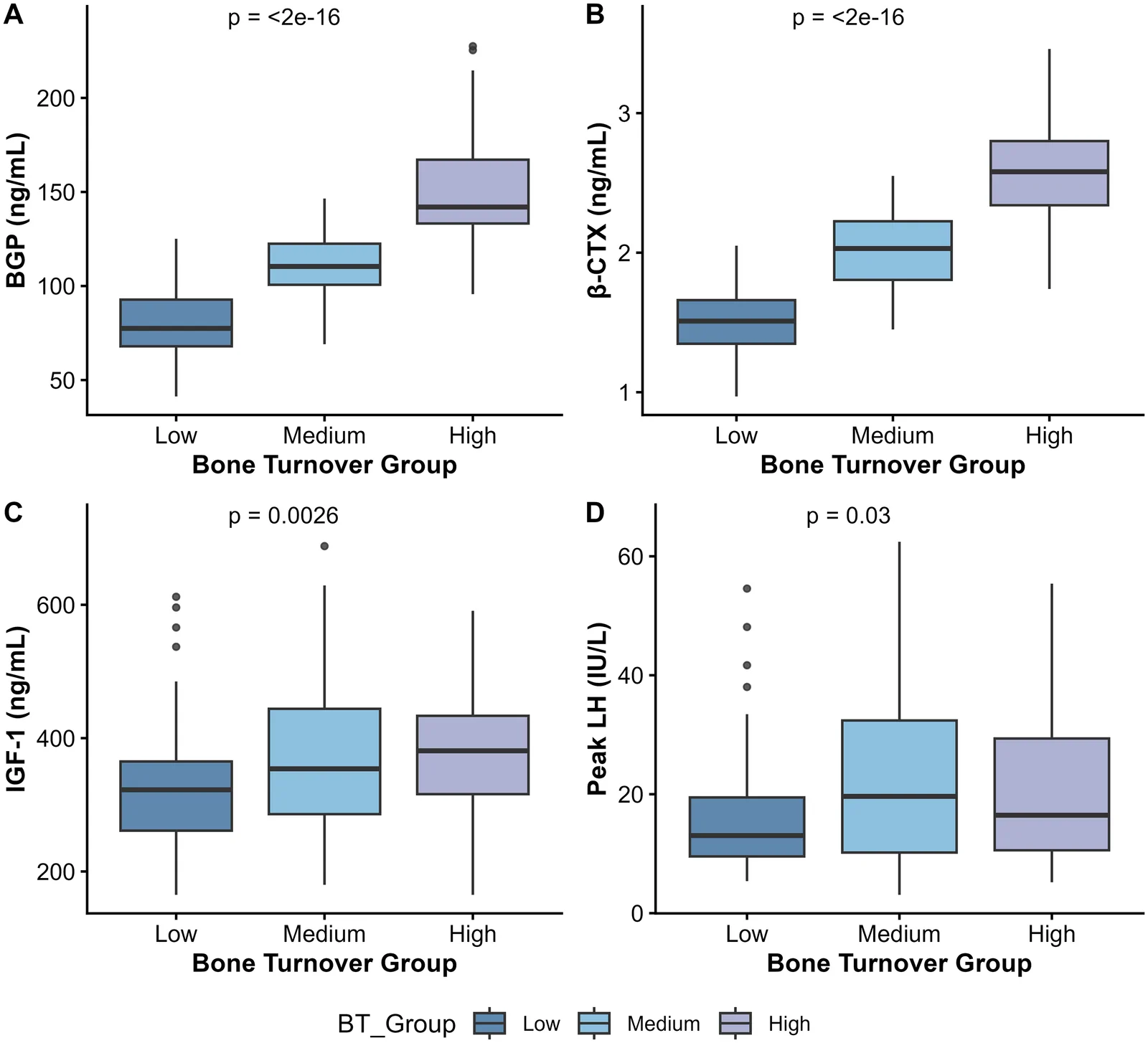
Bone turnover markers and endocrine factors across bone turnover groups in girls with CPP. **(A)** BGP levels. **(B)** β-CTX levels. **(C)** IGF-1 levels. **(D)** Peak LH levels. Box plots show median and interquartile range. Colors indicate bone turnover groups: Low (*n* = 64), Medium (*n* = 63), High (*n* = 63). The omnibus P-values from Kruskal-Wallis test are shown above each plot, and significant increasing trends across groups were confirmed by the Jonckheere-Terpstra test (*p* for trend < 0.001 for BGP and β-CTX; *p* for trend = 0.003 for IGF-1 and 0.030 for peak LH).

The final analysis included 190 girls with CPP. Baseline characteristics of the entire cohort and the bone turnover subgroups are presented in **Table 1**. As designed, both BGP and β-CTX levels showed a significant graded increase across the Low (*n* = 64), Medium (*n* = 63), and High (*n* = 63) bone turnover groups (*p* for trend < 0.001 for both), confirming substantial heterogeneity in bone turnover (**Figures 2A, B**). Bone age advanced progressively with increasing bone turnover (*p* for trend = 0.002), linking higher turnover states to accelerated skeletal maturation. Chronological age also increased slightly but significantly across groups (*p* for trend = 0.008) and was therefore included as a covariate in all subsequent regression models.

**Table 1:** Baseline characteristics of girls with CPP stratified by bone turnover groups.

Variable	Overall (n = 190)	Low (n = 64)	Medium (n = 63)	High (n = 63)	p-value
CA	8.60 (8.00-8.90)	8.40 (7.80-8.83)	8.70 (8.05-8.90)	8.70 (8.40-9.15)	0.008
BA	9.80 (9.00-10.40)	9.40 (8.80-10.12)	9.90 (9.00-10.35)	10.10 (9.50-10.50)	0.002
BGP	110.50 (91.31-134.65)	77.47 (67.95-92.76)	110.40 (100.65-122.50)	142.00 (133.25-167.15)	0.000
β-CTX	2.05 ± 0.54	1.51 ± 0.25	2.02 ± 0.27	2.62 ± 0.37	0.000
IGF-1	347.00 (279.50-419.25)	322.50 (261.25-365.00)	354.00 (286.00-444.00)	381.00 (316.00-433.50)	0.003
IGFBP-3	5.37 ± 0.86	5.24 ± 0.91	5.44 ± 0.87	5.41 ± 0.79	0.376
peak-LH	16.16 (10.07-26.36)	13.05 (9.54-19.46)	19.64 (10.20-32.39)	16.47 (10.58-29.37)	0.030
LH	1.02 (0.46-1.91)	0.82 (0.34-1.30)	1.00 (0.47-1.89)	1.63 (0.69-2.41)	0.008
FSH	4.49 (2.96-6.11)	4.08 (2.48-6.04)	4.48 (3.14-6.10)	5.00 (3.28-6.44)	0.153
E2	95.50 (73.40-163.75)	73.40 (73.40-125.00)	102.00 (73.40-168.50)	121.00 (73.40-176.50)	0.091
TC	4.42 ± 0.81	4.59 ± 0.73	4.35 ± 0.88	4.32 ± 0.79	0.129
TG	0.79 (0.64-1.06)	0.76 (0.67-0.99)	0.86 (0.60-1.08)	0.79 (0.62-1.04)	0.899
FBG	4.93 (4.71-5.15)	4.95 (4.74-5.17)	4.91 (4.70-5.13)	4.92 (4.71-5.09)	0.737
BMDZ	0.90 (0.30-1.90)	0.70 (0.27-1.70)	1.00 (0.15-1.75)	1.10 (0.35-2.30)	0.463

Continuous variables are expressed as mean ± standard deviation or median (interquartile range) based on distribution normality, assessed by the Shapiro-Wilk test. Differences across the three bone turnover groups were compared using one-way ANOVA for normally distributed variables and Kruskal-Wallis tests for non-normally distributed variables. CPP, central precocious puberty; BGP, bone gla protein; β-CTX, β-C-terminal telopeptide of type I collagen.

### IGF-1 and LH peak as independent correlates of bone turnover

**Figure 3:**
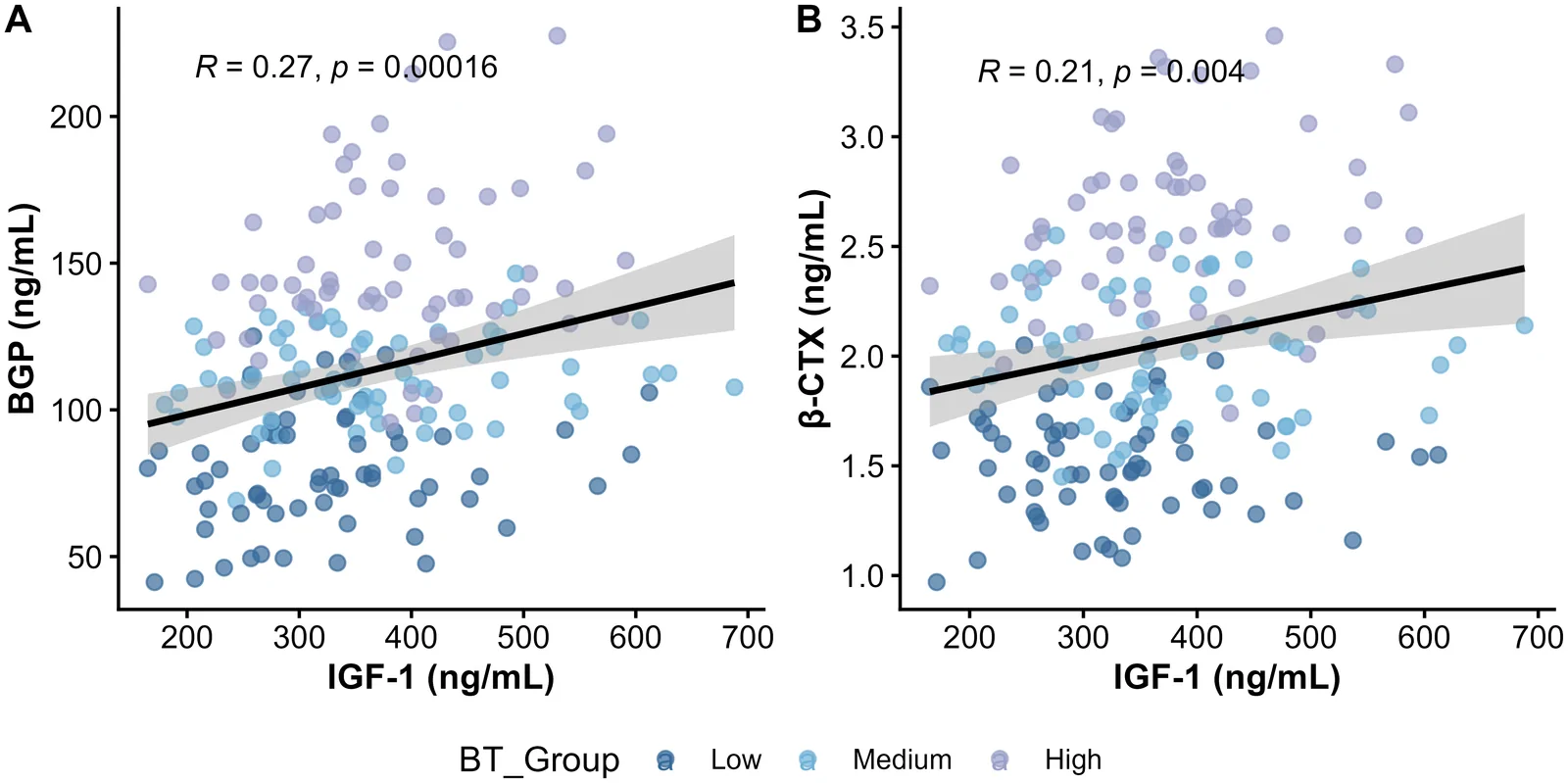
Association between IGF-1 and bone turnover markers. **(A)** Scatter plot of serum IGF-1 levels against BGP levels. **(B)** Scatter plot of serum IGF-1 levels against β-CTX levels. Each point represents an individual participant, colored by bone turnover group. The black line represents the line of best fit from simple linear regression. The strength and significance of the non-parametric correlations were assessed using Spearman’s rank correlation test and are annotated on the plots.

Serum levels of IGF-1 and peak LH increased significantly across the Low, Medium, and High bone turnover groups (*p* for trend = 0.003 and *p*for trend = 0.030, respectively; **Figures 2C, D**). Unadjusted analyses confirmed strong positive correlations of circulating IGF-1 with both bone formation (BGP: Spearman’s *r* = 0.27, *p* = 0.00016) and resorption markers (β-CTX: *r* = 0.21, *p* = 0.004; **Figure 3**). In multiple linear regression models adjusted for chronological age, IGF-1 remained independently associated with both BGP (*β* = 0.069, *p* = 0.005) and β-CTX (*β* = 0.001, *p* = 0.030) (**Table 2**). However, its binding protein, IGFBP-3—analyzed in separate models to avoid multicollinearity—showed no significant association with either BGP (*β* = 4.746, *p* = 0.106) or β-CTX (*β* = -0.008, *p* = 0.854). Regarding the gonadotropic axis, peak LH was associated with BGP (*β* = 0.476, *p* = 0.010; from the IGFBP-3 model) but not with β-CTX (*β* = 0.004, *p* = 0.165), suggesting a preferential link to bone formation. The consistency between bivariate correlations and adjusted models supports a robust relationship between IGF-1 and bone turnover. Notably, we found no significant interaction between IGF-1 and peak LH for either BGP (*p* for interaction = 0.655) or β-CTX (*p* for interaction = 0.791) (**Table 2**), arguing against synergistic activity between the somatotropic and gonadotropic axes in this cohort.

**Table 2:** Multiple linear regression analysis of bone turnover markers.

Model	Dependent	IGF-1 β	IGF-1 p	IGFBP-3 β	IGFBP-3 p	Peak LH β	Peak LH p	Interaction β	Interaction p	CA β	CA p	R²
Model 1a (BGP)	BGP	0.069	0.005			0.411	0.026			8.546	0.007	0.132
Model 1b (BGP)	BGP			4.746	0.106	0.476	0.010			9.950	0.002	0.106
Model 1c (β-CTX)	β-CTX	0.001	0.030			0.004	0.165			0.090	0.066	0.070
Model 1d (β-CTX)	β-CTX			-0.008	0.854	0.005	0.092			0.118	0.016	0.046
Model 2 (BGP)	BGP	0.086	0.058			0.734	0.325	-0.001	0.655	8.632	0.006	0.132
Model 2 (β-CTX)	β-CTX	0.001	0.340			0.001	0.931	0.000	0.791	0.090	0.070	0.070

Models were adjusted for chronological age. Model 1a and 1c assessed the main effects of IGF-1 and peak LH. Model 1b and 1d assessed the main effects of IGFBP-3 and peak LH in separate models to avoid multicollinearity. Model 2 included the interaction term (IGF-1 × LH peak). BGP, bone gla protein; β-CTX, β-C-terminal telopeptide of type I collagen.

### Specificity of associations and exploratory analyses

The associations of IGF-1 and LH peak with bone turnover were specific. In contrast, no significant differences in any glucolipid metabolic parameters​ (TC, TG, HDL-C, LDL-C, FBG) were observed across the bone turnover groups (**Table 1**, *p* for trend > 0.05 for all). This pattern indicates that the heterogeneity in bone turnover is primarily related to the somatotropic and gonadal axes rather than to general metabolic status.

An exploratory analysis revealed an inverse correlation between serum HDL-C and lumbar spine bone mineral density Z-score (BMD_Z_) (*r* = -0.20, *p* = 0.0047; **Supplementary Figure 1B**). This finding should be interpreted with caution as it was exploratory and not adjusted for potential confounders like body composition.

The robustness of the primary findings was supported by sensitivity analyses. The association of IGF-1 with BTMs was consistent across age subgroups (**Supplementary Table 1**). Furthermore, E-value analysis suggested that unmeasured confounding was unlikely to fully explain the observed effect sizes (**Supplementary Table 2**). The study’s sample size of 190 provided sufficient statistical power to detect the observed effect sizes, as evidenced by the significant independent associations of IGF-1 with BTMs (**Table 2**).

## Discussion

This cross-sectional study investigates the drivers of bone turnover heterogeneity at diagnosis in a cohort of treatment-naïve girls with CPP. Our data show that serum IGF-1 levels are strongly and independently associated with both bone formation and resorption markers, while peak LH shows a more selective association. Critically, we found no evidence of a synergistic interaction between IGF-1 and peak LH. These observations suggest that, in this pathological state of accelerated puberty, the somatotropic axis might operate as a predominant, independent driver of bone turnover heterogeneity, a perspective that diverges from the established physiological model of somatotropic-gonadal synergy during normal puberty (31, 32). Furthermore, the lack of association with glucolipid metabolic parameters underscores the potential specificity of this neuroendocrine association.

### IGF-1 as a strong, independent correlate of bone turnover

Our findings position IGF-1 as a strong, independent correlate of bone turnover heterogeneity in CPP. The significant association of IGF-1, but not its binding protein IGFBP-3, with bone turnover markers suggests that the bioactivity of IGF-1—governed by its free fraction—is a critical correlate of bone metabolism. This is evidenced by the graded increase in IGF-1 across bone turnover groups and its independent associations with both bone formation (BGP) and resorption (β-CTX), a pattern consistent with the pleiotropic actions of IGF-1 (33). This dissociation aligns with observations that IGF-1 is a more dynamic marker of growth velocity than IGFBP-3 in pubertal disorders (34). Mechanistically, IGF-1 acts as a mitogen for osteoblast precursors, directly stimulating bone matrix synthesis, while concurrently promoting osteoclastogenesis via the RANKL pathway, thereby coupling bone resorption to formation (35–37). This dual action could explain the synchronized elevation of BGP and β-CTX, resulting in the high-bone-turnover state observed (38). These findings are further supported by preclinical models demonstrating that IGF-1 signaling is indispensable for normal bone formation and accrual (35, 36).

### Gonadotropin action: an indirect and selective role

In contrast to the broad, direct effects of IGF-1, peak LH exhibited a selective association—correlating only with the bone formation marker BGP but not with β-CTX. This selectivity underscores the indirect nature of gonadotropin actionon bone, as LH receptors are not expressed on bone cells; thus, LH likely influences bone metabolism primarily via stimulating ovarian estrogen synthesis. Estrogen is well-characterized to suppress bone resorption and promote epiphyseal fusion (39, 40). The isolated association with BGP in our cohort may reflect an estrogen-mediated, indirect stimulation of bone formation—potentially through upregulating the GH/IGF-1 axis (41, 42). As our regression models adjusted for IGF-1, this indirect effect may have been accounted for, leaving a residual association that warrants further mechanistic investigation. This finding is consistent with variable effects of gonadotropin-releasing hormone agonist (GnRHa) therapy on bone mineral density (BMD) in CPP, underscoring the complexity of the gonadal axis’s contribution to bone metabolism (43). Similarly, serum FSH levels did not differ significantly across bone turnover groups (**Table 1**), arguing against a major contribution of FSH to the observed bone turnover heterogeneity in this early CPP cohort.

### Reconceptualizing pubertal bone regulation: from synergy to somatotropic dominance

Taken together, our results depicting a dominant role for IGF-1 and a selective, indirect role for LH prompt a reconceptualization of pubertal bone regulation in CPP. The well-established model of somatotropic-gonadal synergy posits that estrogen potentiates GH secretion, which upregulates IGF-1 production, creating a feed-forward loop that maximizes bone accrual during puberty (32). Our results, however, indicate a critical departure from this model in CPP, as evidenced by the non-significant interaction between IGF-1 and peak LH, which suggests a disruption of their coordinated regulation. Given that LH exerts its skeletal effects indirectly via estrogen, this uncoupling supports a shift toward somatotropic dominance.

We therefore propose a “somatotropic-dominant” model​ for bone remodeling in CPP. This model posits that the potent, direct anabolic effects of IGF-1 on the skeleton are primarily driven by the central activation of the hypothalamic-pituitary axis that defines CPP, rather than being solely dependent on gonadal steroid-mediated amplification. This perspective finds a parallel in McCune-Albright syndrome, where GH excess independently drives skeletal maturation alongside precocious puberty, demonstrating the potent, discrete capacity of the somatotropic axis to regulate bone (44, 45). While the etiology differs, this parallel underscores the principle that somatotropic drive can operate as a powerful, semi-autonomous force in skeletal growth.

Critically, this “somatotropic-dominant” model is consistent with the underlying neuroendocrine dysregulation in CPP. The central kisspeptin-neurokinin B signaling pathway, which triggers the GnRH pulse generator, is also intricately linked to the regulation of GH secretion (46–48). Thus, the elevated IGF-1 levels and consequent bone phenotype we observe may represent a co-manifestation of a pervasive central activation, with IGF-1 acting as the key peripheral effector directly driving bone remodeling. This provides a plausible neuroendocrine framework for our observed departure from the classic synergy model.

### Neuroendocrine specificity and clinical translation

A key strength of our study is the concurrent evaluation of glucolipid metabolism, which revealed no significant association with bone turnover heterogeneity. This finding argues against a primary role for general metabolic status and instead underscores the specificity of the somatotropic axis in mediating bone turnover regulation in CPP.​ An exploratory inverse correlation between HDL-C and lumbar spine BMD_Z_ was observed; however, this isolated finding was unadjusted for body composition—a key confounder—and should be interpreted with caution (49). It does not undermine our primary conclusion that dynamic bone turnover is dissociated from glucolipid metabolism in this context.

These cross-sectional insights may have clinical implications.​ Current CPP management focuses on suppressing the gonadal axis with GnRHa to decelerate skeletal maturation (4, 21). Our data suggest that​ integrating IGF-1 as a potential​ biomarker for risk stratification could be considered.​ Girls with CPP and elevated IGF-1 levels—potentially​ indicative of a high-turnover state—might​ benefit from more vigilant monitoring (e.g., serial BA assessments). The robustness of the IGF-1-BTM association, consistent across sensitivity analyses, lends support to​ its potential​ clinical utility. However, the prognostic value of baseline IGF-1 for longitudinal growth outcomes needs to be confirmed in future studies.

## Limitations

First and foremost, the retrospective, cross-sectional design precludes any causal inference regarding the observed associations and cannot capture longitudinal changes in bone markers, growth velocity, or final adult height. Our findings represent a phenotypic snapshot at diagnosis and should be interpreted as hypothesis-generating.​ Second, the absence of data on pubertal Tanner stage, body mass index, and body composition limits our ability to fully adjust for these important confounders of bone metabolism and IGF-1 levels; however, E-value analysis suggested that residual confounding by such unmeasured factors is unlikely to fully account for the observed effect sizes. Third, the use of peak LH as the quantitative proxy for gonadal axis activity represents a limitation of this study. Although peak LH is the gold standard for confirming HPG axis activation in CPP, it does not act directly on bone tissue; its association with bone turnover reflects downstream ovarian estrogen secretion. We did measure serum E2 in all 190 participants (**Table 1**); however, E2 levels showed no significant gradient across the Low, Medium, and High bone turnover groups (*p* for trend = 0.091). This lack of a clear dose-response relationship is likely attributable to the known volatility and physiological fluctuations of single-point E2 measurements in early CPP. Future studies should incorporate serial or pooled estradiol assessments to more directly evaluate gonadal steroid effects on bone remodeling. Fourth, we measured BGP rather than the International Osteoporosis Foundation (IOF)−recommended P1NP (Procollagen type I N−terminal propeptide) as the bone formation marker due to the retrospective reliance on routine clinical assays. Although BGP is a validated indicator of osteoblastic activity in children, future prospective studies should incorporate P1NP to confirm our findings. Fifth, as noted in the Methods, this study focused on a homogeneous cohort of girls with biochemically confirmed CPP and did not include a control group (e.g., girls with isolated premature thelarche or age-matched healthy girls). Therefore, we cannot determine whether the observed associations are specific to CPP or a general feature of pubertal acceleration. Future prospective, longitudinal studies incorporating Tanner staging, anthropometric measures, and control groups are needed to confirm the temporal relationships and specificity of the associations observed here.​ Sixth, fracture data were not collected, precluding an assessment of how bone turnover heterogeneity relates to skeletal fragility in this population. Seventh, bone age assessment, despite being performed by a blinded expert, has inherent subjectivity. Finally, although measured, key bone regulators (e.g., 25-hydroxyvitamin D, parathyroid hormone) were omitted from the primary analysis to maintain focus on the somatotropic-gonadal axis interplay, a strategic choice that does not invalidate the core findings.

## Conclusions

In conclusion, this cross-sectional study demonstrates that marked heterogeneity in bone turnover among girls with CPP at diagnosis is strongly and independently associated with the somatotropic axis, as reflected by serum IGF-1 levels. We found no evidence of synergy between IGF-1 and gonadotropin activity. These findings challenge the classical paradigm of somatotropic-gonadal axis synergy in pubertal bone acquisition and posit IGF-1 as a potential key biomarker for skeletal metabolism assessment in CPP. The proposed somatotropic-dominant mechanism requires validation in longitudinal studies.

## Data Availability

The raw data supporting the conclusions of this article will be made available by the authors, without undue reservation.
