# Correction: Somatotropic axis drives bone turnover heterogeneity independently of gonadotropin synergy in girls with central precocious puberty

**DOI:** 10.3389/fendo.2026.1952761

**Published:** 2026-08-11

**Authors:** Fuhui Liu, Haiqin Jiang, Zhenzhen Li, Lei Li

**Affiliations:** 1 School of Clinical Medicine, Shandong Second Medicine University, Weifang, China; 2 Department of Geriatrics I, Weifang People’s Hospital, Weifang, China; 3 Department of Pediatrics, Weifang People’s Hospital, Weifang, China

**Keywords:** bone turnover markers, central precocious puberty, insulin-like growth factor I, luteinizing hormone, somatotropic axis

Affiliation “³Department of Pediatrics, Weifang People’s Hospital, Weifang, China” was omitted from the paper. This affiliation has now been added for author(s) “Zhenzhen Li and Lei Li”.

Affiliation “²Department of Geriatrics I, Weifang People’s Hospital, Weifang, China” was erroneously included in the paper. This affiliation has now been removed for author(s) “Zhenzhen Li and Lei Li”.

The original version of this article has been updated.

