# Supplementary material for: Somatotropic axis drives bone turnover heterogeneity independently of gonadotropin synergy in girls with central precocious puberty
Source: Front Endocrinol (Lausanne). 2026 Jul 22;17:1858179. doi: 10.3389/fendo.2026.1858179 (PMC13437337; doi:10.3389/fendo.2026.1858179)
Supplement: Supplementary file 1 [file DataSheet1.pdf]

Supplementary Information

Figures: 1; Tables: 2

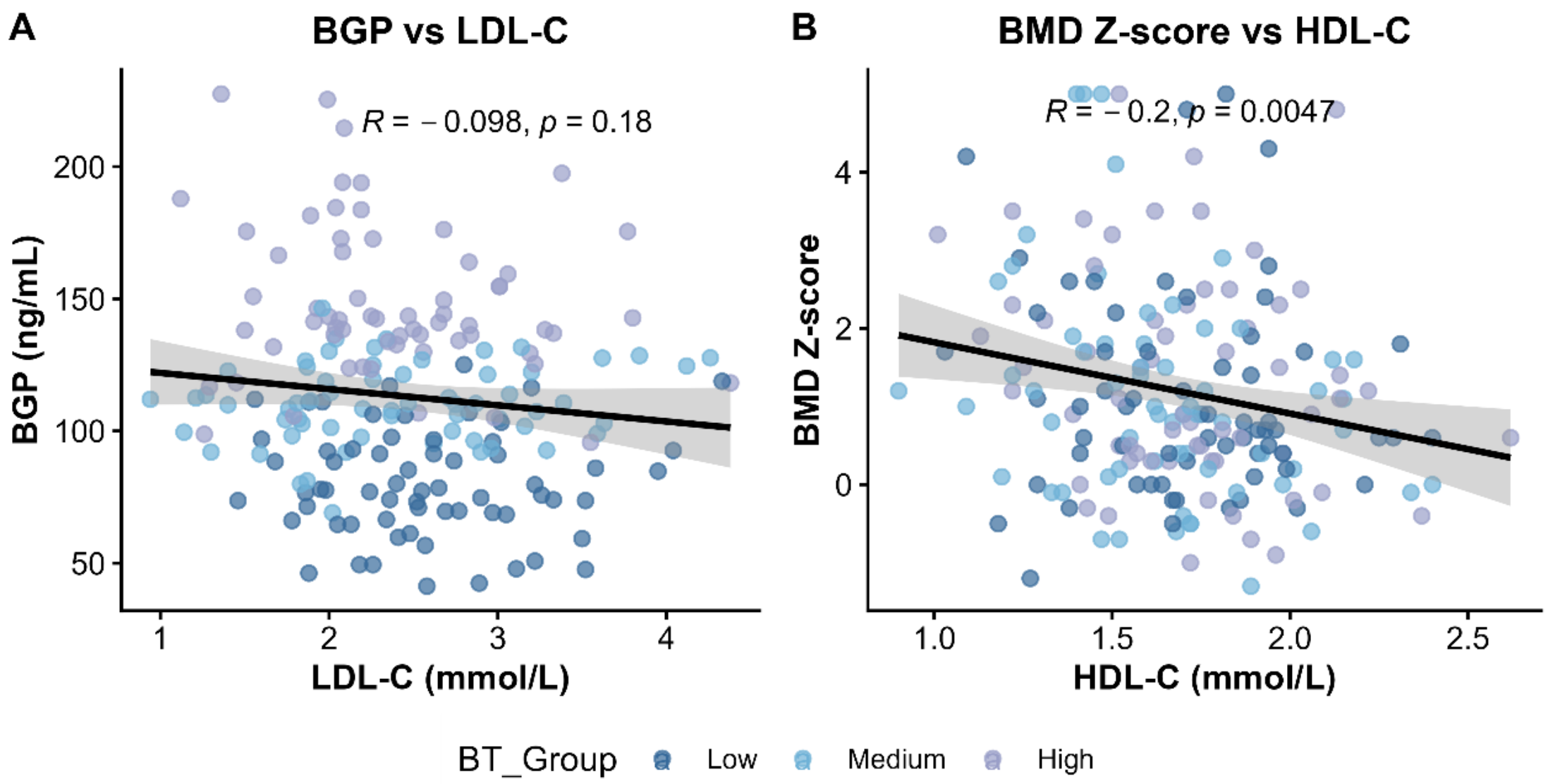

Supplementary Figure 1. Exploratory analyses of associations between glucolipid metabolic parameters and bone measures.

(A) Scatter plot of LDL-C levels against BGP levels. (B) Scatter plot of HDL-C levels against the lumbar spine bone mineral density Z-score (BMD Z-score).

Statistical analysis was performed using Spearman's rank correlation test. No significant correlation was observed between LDL-C and BGP ($p = 0.18$). A weak but significant inverse correlation was found between HDL-C and BMD Z-score ($r = -0.20$, $p = 0.0047$). The association in panel B was exploratory and should be interpreted with caution.

Supplementary Table 1. Age-Stratified Analysis of IGF-1 and LH Peak Associations with Bone Turnover Markers

Age_Group	Variable	BGP *β*	BGP *p*	β-CTX *β*	β-CTX *p*
Younger	IGF-1	0.074	0.026	0.001	0.081
Younger	peak LH	0.640	0.012	0.004	0.304
Older	IGF-1	0.079	0.026	0.001	0.077
Older	peak LH	0.147	0.594	0.003	0.389

Analysis stratified by median chronological age. Coefficients from linear regression models (IGF-1 and LH peak as independent variables, bone turnover markers as dependent variables).

Supplementary Table 2. E-value Sensitivity Analysis for Unmeasured Confounding

Parameter	Value	Interpretation
Observed association (Risk Ratio)	1.0018	Strength of association between IGF-1 and BGP
E-value for point estimate	1.04	Minimum strength of unmeasured confounding to explain away the observed association
E-value for confidence interval limit	1.02	Minimum strength of unmeasured confounding to shift CI to include the null

E-values quantify the minimum strength of association that an unmeasured confounder would need to have with both the exposure (IGF-1) and outcome (BGP) to explain away the observed association. An E-value of 1.04 indicates that an unmeasured confounder would need to be at least weakly associated with both IGF-1 and BGP ($RR \geq 1.04$) to fully explain the observed effect. Given that strong established risk factors (e.g., BMI) typically have much larger effect sizes, this small E-value supports the robustness of the association against potential unmeasured confounding. Analysis based on Model 1a (BGP ~ IGF1 + LH_peak + CA).
